# Supplementary figures and images for: m6A Methylation-Mediated Stabilization of LINC01106 Suppresses Bladder Cancer Progression by Regulating the miR-3148/DAB1 Axis
Source: Biomedicines. 2024 Jan 5;12(1):114. doi: 10.3390/biomedicines12010114 (PMC10813768; doi:10.3390/biomedicines12010114)

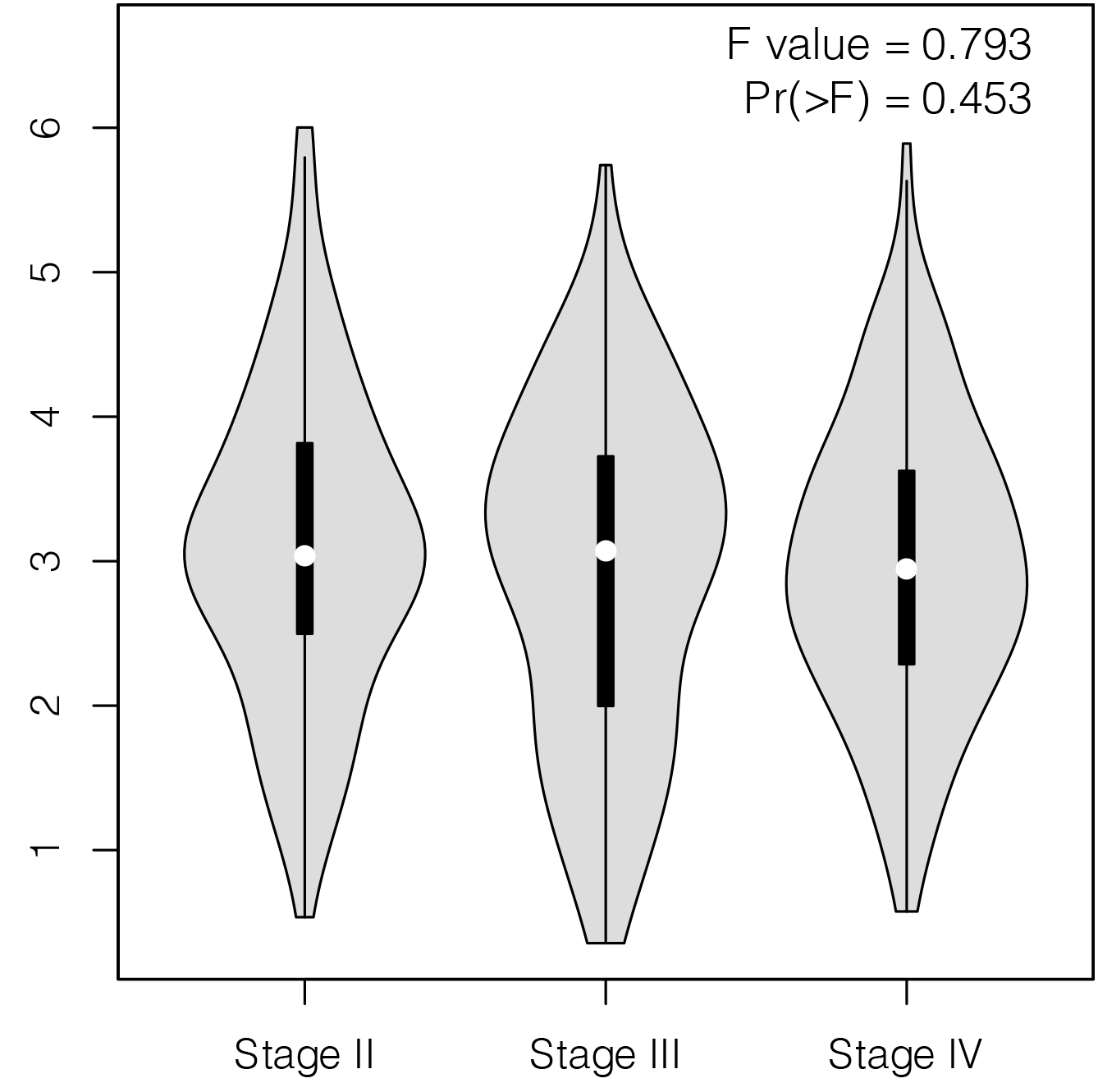

Supplement: Supplementary file 1 [file biomedicines-12-00114-s001.zip › Supplementary Files/Figure S1.tif]

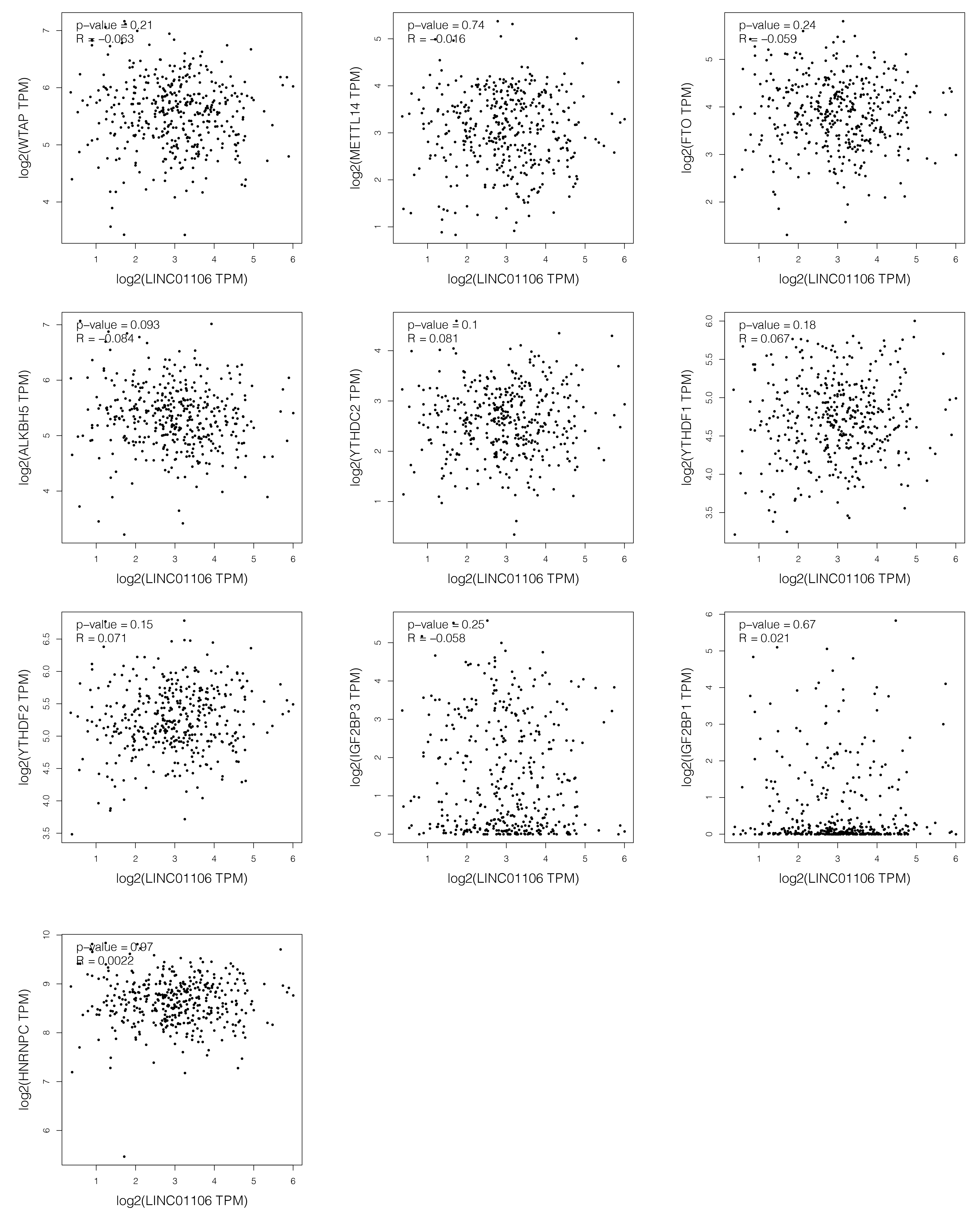

Supplement: Supplementary file 1 [file biomedicines-12-00114-s001.zip › Supplementary Files/Figure S2.jpg]
